# Effect of a School-Based Educational Intervention About the Human Papillomavirus Vaccine on Psychosocial Outcomes Among Adolescents: Analysis of Secondary Outcomes of a Cluster Randomized Trial

**DOI:** 10.1001/jamanetworkopen.2021.29057

**Published:** 2021-11-02

**Authors:** Cristyn Davies, Helen S. Marshall, Gregory Zimet, Kirsten McCaffery, Julia M. L. Brotherton, Melissa Kang, Suzanne Garland, John Kaldor, Kevin McGeechan, S. Rachel Skinner

**Affiliations:** 1 Specialty of Child and Adolescent Health, Faculty of Medicine and Health, The University of Sydney, Sydney, New South Wales, Australia; 2 Women’s and Children’s Hospital and School of Medicine and Robinson Research Institute, The University of Adelaide, South Australia, Australia; 3 Department of Pediatrics, Division of Adolescent Medicine, Indiana University School of Medicine, Indianapolis; 4 Sydney Health Literacy Lab, School of Public Health, Faculty of Medicine and Health, The University of Sydney, Sydney, New South Wales, Australia; 5 VCS Population Health, VCS Foundation, Victoria, Australia; 6 Melbourne School of Population and Global Health, The University of Melbourne, Melbourne, Victoria, Australia; 7 Specialty of General Practice, Faculty of Medicine and Health, The University of Sydney, Sydney, New South Wales, Australia; 8 Faculty of Health, School of Public Health, University of Technology Sydney, Sydney, New South Wales, Australia; 9 Centre for Women’s Infectious Diseases Research, The Royal Women’s Hospital, Melbourne, Victoria, Australia; 10 Reproductive and Neonatal Infectious Diseases, Department of Obstetrics and Gynaecology, University of Melbourne, Murdoch Children’s Research Institute, Melbourne, Victoria, Australia; 11 The Kirby Institute for Infection and Immunity in Society, Faculty of Medicine, UNSW Sydney, Sydney, New South Wales, Australia; 12 Faculty of Medicine and Health, The University of Sydney School of Public Health, Sydney, New South Wales, Australia

## Abstract

**Importance:**

Delivery of vaccination to adolescents via a school-based program provides an opportunity to promote their involvement in health decision-making, service provision, and self-efficacy (belief in one’s ability to perform a certain behavior).

**Objective:**

To examine the effect of a human papillomavirus (HPV) vaccination education and logistical intervention on adolescent psychosocial outcomes.

**Design, Setting, and Participants:**

In this cluster randomized trial and process and qualitative evaluation, adolescents aged 12 to 13 years (first year of high school) were recruited at high schools in Western Australia (WA) and South Australia (SA) in 2013 and 2014. Statistical analysis was performed from January 2016 to December 2020.

**Interventions:**

The complex intervention consisted of an adolescent intervention to promote knowledge and psychosocial outcomes, shared decisional support tool, and logistical strategies.

**Main Outcomes and Measures:**

Prespecified secondary outcomes were assessed. The HPV Adolescent Vaccination Intervention Questionnaire (HAVIQ) was used to measure changes in adolescent knowledge (6-item subscale), fear and anxiety (6-item subscale), self-efficacy (5-item subscale), and decision-making (8-item subscale). The hypothesis was that the intervention would improve adolescent involvement in vaccine decision-making (measured before dose 1 only), improve vaccine-related self-efficacy, and reduce vaccine-related fear and anxiety (measured before doses 1, 2, and 3). Mean (SD) scores for each subscale were compared between intervention and control students. In the process evaluation, focus groups were conducted. Analyses of the HAVIQ data were conducted from 2016 to 2020. Qualitative analyses of the focus groups were undertaken from 2017 to 2020.

**Results:**

The trial included 40 schools (21 intervention and 19 control) across sectors with 6967 adolescents (mean [SD] age, 13.70 [0.45] years). There were 3805 students (1689 girls and 2116 boys) in the intervention group and 3162 students (1471 girls and 1691 boys) in the control group. The overall response rate for the HAVIQ was 55%. In WA, where parental consent was required, the response rate was 35% (1676 of 4751 students); in SA, where parental consent was not required, it was 97% (2166 of 2216 students). The mean (SD) score for decision-making in the intervention group before dose 1 was 3.50 (0.42) of 5 points and 3.40 (0.40) in the control group, a small but significant difference of 0.11 point (95% CI, 0.06 to 0.16 point; *P* < .001). There was a small difference in favor of the intervention group in reduced vaccination-related anxiety (pre–dose 1 difference, −0.11 point [95% CI, −0.19 to −0.02 point]; pre–dose 2 difference, −0.18 point [95% CI, −0.26 to −0.10 point]; pre–dose 3 difference, −0.18 [95% CI, −0.24 to −0.11]) and increased vaccination self-efficacy (pre–dose 1 difference, 4.0 points; [95% CI, 1.0 to 7.0 points]; pre–dose 2 difference, 4.0 points [95% CI, 2.0 to 6.0 points]; pre–dose 3 difference, 3.0 points [95% CI, 1.0 to 5.0 points]). Focus group data from 111 adolescents in 6 intervention and 5 control schools revealed more confidence and less anxiety with each vaccine dose.

**Conclusions and Relevance:**

In this cluster randomized trial, there was a small difference in adolescent decisional involvement and vaccine-related confidence and reduced vaccination-related fear and anxiety that was maintained throughout the vaccine course in the intervention vs control groups. Guidelines for vaccination at school should incorporate advice regarding how this outcome can be achieved.

**Trial Registration:**

Australian and New Zealand Clinical Trials Registry: ACTRN12614000404628

## Key Points

**Question:**

Does a human papillomavirus (HPV) vaccination intervention in the school setting improve adolescent psychosocial outcomes?

**Findings:**

In this analysis of secondary outcomes of a cluster randomized trial and qualitative evaluation of an education and logistical intervention (HPV.edu) to improve acceptability of HPV vaccination in 40 schools (6967 adolescents), the mean score for adolescent involvement in decision-making was significantly higher in the intervention group than in the control group. There were also small, significant differences in favor of the intervention group with respect to adolescents’ vaccination-related anxiety and self-efficacy scores, measured before each of the 3 vaccine doses.

**Meaning:**

An HPV vaccination intervention implemented in schools improved psychosocial vaccine-related outcomes in adolescents, an effect that was maintained throughout the vaccination course.

## Teaser

This analysis of secondary outcomes of a cluster randomized trial examines the effect of a human papillomavirus (HPV) vaccination education and logistical intervention on adolescent psychosocial outcomes.

## Introduction

Delivery of vaccination to adolescents via a school-based program provides an opportunity to promote their involvement in health decision-making, service provision, and self-efficacy (belief in one’s ability to perform a certain behavior). This involvement, in turn, assists in the development of adolescent autonomy and vaccination literacy and can enhance the quality of health services.^1,2,3,4^ Negative experiences may lead to vaccination-related fear and anxiety, refusal on vaccination day, or delays or avoidance of vaccination in the future.^5,6,7^ Implementing fear and pain mitigation strategies is considered good immunization practice.^5,8,9^ Hesitancy toward vaccination associated with needle-related pain may also result in suboptimal uptake.^5,10^ Fear of needles, which may or may not be related to pain, is a common experience in adolescent vaccination programs, and syncope is a relatively common adverse event that is of special interest with regard to human papillomavirus (HPV) vaccination, as with other adolescent vaccines.^8,11,12^ The World Health Organization has developed the term *immunization stress-related response* to describe signs and symptoms that may arise around the time of immunization related to patient anxiety, but not to the vaccine product, a defect in its quality, or an error in its administration.^8,13^

Parents typically have primary responsibility for decision-making for HPV vaccination, particularly in clinical settings.^6^ However, it is important to examine the role of adolescents in vaccination decision-making in nonclinical settings such as schools. We have shown that adolescents’ HPV vaccination understanding, involvement in decision-making, and self-efficacy are low and their vaccination-related fear and anxiety are high in the school program setting.^14,15^ Adolescent agency and self-efficacy are worthy outcomes because they enhance adolescent vaccination literacy and can help to improve the vaccine experience. There is limited research that examines pain and fear mitigation interventions for adolescent vaccination, including effective management strategies in mass campaigns and school program settings, and the impact of settings in which vaccines are administered.^5,16,17,18,19,20,21,22,23,24^

In Australia, HPV vaccination commenced in 2007 for girls aged 12 to 13 years in the first year of high-school using a 3-dose schedule of the quadrivalent HPV vaccine and was extended to male adolescents in 2013. From 2018, the program transitioned to the 9-valent HPV vaccine in a 2-dose schedule.^25^ In 2013 and 2014, we conducted a multicenter cluster randomized clinical trial (HPV.edu) in 2 Australian states.^26^ HPV.edu aimed to examine the impact of a complex education and logistical intervention on student knowledge about HPV vaccination (vaccination literacy),^27^ psychosocial outcomes, and vaccine uptake. Intervention schools administered an adolescent intervention (education and distraction on the day),^28^ a shared decisional support tool (DST) for parents and adolescents, and logistical strategies described later in this article.

The primary outcome of HPV.edu was the difference in 3-dose HPV vaccination completion rates between intervention and control schools. Secondary outcomes included (1) the difference in scores of the HPV Adolescent Vaccine Intervention Questionnaire (HAVIQ)^29^ between students in intervention and control schools before each HPV vaccine dose and (2) the difference between the time to vaccinate students in intervention and control groups and the difference in proportion of consent form returns between intervention and control schools.^1^

We have published the primary outcome of the difference in HPV dose 1 uptake across intervention and control schools.^27^ There was no significant difference. We plan to subsequently report the full primary outcome of the difference in 3-dose uptake between intervention and control schools, with secondary outcome 2, and a full implementation evaluation. We have previously reported the secondary outcome of difference in HPV vaccination knowledge and attitudes between students in intervention and control schools, showing gains in student knowledge.^27^ We have also previously reported findings that parents had positive attitudes toward school-based vaccination for adolescents and that HPV vaccine decision-making for parents was influenced by school-based vaccination.^30^

In this study, we sought to answer the question of whether an HPV vaccination intervention in the school setting improves adolescent psychosocial outcomes (secondary outcome 1). We hypothesized an improvement in adolescent involvement in vaccine decision-making, self-efficacy with vaccination, and reduced vaccination-related fear and anxiety.

## Methods

In Australia, HPV vaccination is administered free to eligible adolescents at school through the National Immunisation Program. Adolescents are primarily vaccinated en masse, on school grounds, after parental consent is obtained.^27^ We obtained ethical approval from the Department of Health Western Australia Human Research Ethics Committee, Women’s and Children’s Hospital Human Research Ethics Committee, relevant government authorities, and the University of Sydney, Australia. Parents provided consent for adolescent participation in 1 state (South Australia [SA]) for the HAVIQ; all parents provided consent for their adolescent to participate in the process evaluation, and adolescents provided verbal assent. Our advisory board had representatives from the health department and immunization teams in Western Australia (WA) and SA, and the government, Catholic school, and independent education sectors. The board provided input on all aspects of the study. We followed the Consolidated Standards of Reporting Trials (CONSORT) reporting guidelines^29^ for reporting on clinical trials in this study.

### Design

Our study was a community-based, cluster randomized trial. Our trial protocol has been published elsewhere^26^ and is provided in Supplement 1. In this study, we recruited schools (clusters) with year 8 enrollments of more than 100 students. Eligible schools were stratified by educational sector (independent, Catholic, or government) and state (WA and SA). Schools were randomly selected and invited to participate. We randomly allocated schools (1:1) to intervention or control.^26,27^ We also undertook a process evaluation, including a qualitative study. HPV vaccination for adolescent boys was introduced in 2013, with slightly different timelines across states. Therefore, only single-gender schools were recruited in 2013 and coeducational schools in 2014. The intervention and data collection spanned 2013 to 2015. We undertook analyses of the HAVIQ data from 2016 to 2020. Qualitative analyses of focus group interviews were undertaken from 2017 to 2020. To guide our approach, analyses, and interpretation of data, we used an ecological framework to understand multiple levels of influence within a complex system, including the psychological, social, and organizational levels of influence on parents within an adolescent school vaccination system.

### Changes to Protocol After Publication

We originally intended to use an HPV vaccination decision aid for parents and adolescents to use together. After HPV.edu study advisory board guidance, this was modified to a shared DST, a more succinct resource promoting vaccination for parents and adolescents to use together with accessible language and diagrams. Advisory board guidance precluded nonmonetary incentives for consent form return and additional in-school vaccination catch-up visits.

### Participants and Recruitment

Principals provided consent for the school to participate; in 1 state, teachers involved in student data collection also provided consent to participate in study activities. Schools provided study information to eligible students. Each state’s ethics committee provided a waiver of parental consent for adolescent participation in the intervention. One state’s ethics committee approved a consent waiver for student data collection, and the other state required explicit parental consent. Student assent was obtained for data collection.

### Intervention

Control schools conducted the vaccination program as per their usual practice. Intervention schools delivered a multicomponent education and logistical intervention comprising (1) an adolescent intervention^28^ (education and distraction) to promote adolescent knowledge, decision-making involvement, and confidence in vaccination and to reduce vaccine-related anxiety; (2) a DST; and (3) logistical strategies for the school, including consent form return strategies and vaccination-day guidelines for setup of the vaccination room.^26,27,28^ The DST was sent home with adolescents at the same time as the vaccination consent form.^26^ Logistical strategies for immunization teams were designed to improve vaccination uptake (not reported here), school vaccination processes, and adolescent experience. They included guidelines^26^ about optimal vaccination room setup to minimize student anxiety, promote student privacy, and assist with the efficiency of vaccination processes and distraction strategies to directly assist in management of adolescent anxiety. Study staff offered training and information about the interventions to school personnel and their respective immunization teams. More details on how the intervention was administered have been published elsewhere.^27^

### Outcomes

Study outcomes included differences in adolescent vaccination-related psychosocial outcomes, including decision-making, fear and anxiety, and self-efficacy. We developed and validated the HAVIQ^30^ to collect data in 4 subscales: (1) knowledge about HPV and the HPV vaccine (6 items), (2) adolescent involvement in decision-making (8 items), (3) fear and anxiety associated with the vaccine (6 items), and (4) self-efficacy in receiving the vaccine (5 items).^30^ The HAVIQ development and validation occurred in a number of stages: item development, measure development, and assessment of face validity, content validity, and internal reliability. After this process, content validity for each measure was greater than minimum requirements. For assessment of internal reliability, Cronbach α values were calculated for each measure: knowledge (α = 0.6), fear and anxiety (α = 0.79), self-efficacy (α = 0.79), and decision-making (α = 0.35). Although the last value was low, item correlation was good, with no evidence of multicollinearity. Intervention impact was evaluated using a pretest and posttest of mean difference in scores for each relevant subscale on the measure, between intervention and control groups.^30^ The HAVIQ was administered by school personnel after students participated in the education intervention for the intervention group and before HPV vaccine dose 1 in both intervention and control groups. Students then completed the components of HAVIQ before HPV vaccine doses 2 and 3 to measure change in scores after experience with vaccination (eTable 1 in Supplement 2).

In this study, we present results for subscales 2 to 4, which relate to adolescent vaccination experience and psychosocial outcomes. The findings for knowledge, subscale 1, have been published elsewhere.^30^

### Sample Size

Sample size was calculated to detect an increase in vaccination uptake from 70% to 80% at .05 significance and with a power of 80%, assuming an intraclass correlation coefficient of 0.05.^26^ To allow for dropout, we increased the sample by 10% and aimed to recruit a total of 40 schools.^26^

### Randomization

We recruited a stratified random sample of schools, which we then randomly allocated to intervention and control groups.^26^ This process and that of blinding, took place as per the protocol.^26^

### Statistical Analysis

Mean scores of self-efficacy, fear and anxiety, and decision-making were compared between intervention and control groups at each time point using 2-sample *t* tests with appropriate adjustment for clustering. The proportion of consent form returns were compared between groups before dose 1 using χ^2^ tests with appropriate adjustment for clustering. Significance was set at *P* < .05. Statistical analyses were performed with SAS statistical software version 9.4 (SAS Institute).

We conducted a process evaluation (2013-2015) from a sample of 11 schools (5 control and 6 intervention) participating in the main study (HPV.edu). Six intervention and 6 control schools were recruited; however, 1 control school participated in the observations only because of their busy schedule at the time. In semistructured focus groups, adolescents were asked about HPV-related knowledge, vaccination decision-making, and confidence related to vaccination. Student focus groups took place after HPV dose 2 or 3 had been offered in the school program. Focus groups were digitally recorded and transcribed verbatim. Recruitment stopped once data saturation (or data adequacy) was achieved.

The first author (C.D.) performed all analyses and was blinded to study group (intervention or control) at all times. Transcripts were coded in NVivo9 qualitative data analysis software version 9 (QSR International), and data were subjected to thematic analysis.^31,32^ Inductive and deductive approaches were used to generate codes used across the data set. C.D. developed codes with input from the research team and 2 student assistants: coding was undertaken sentence by sentence, identifying and discussing themes. Conceptual saturation was reached when no new codes were generated. C.D. performed an overall analysis to ensure that diverse themes emerging from the data set were represented. Data analysis was performed from January 2016 to December 2020.

## Results

There were 6967 students (mean [SD] age, 13.70 [0.45] years) in our 40 study schools (21 intervention and 19 control). There were 3805 students (1689 girls, 2116 boys) in the intervention group and 3162 students (1471 girls, 1691 boys) in the control group. Two-thirds were recruited in WA, and one-third were recruited in SA. Approximately one-half of the students were recruited from government schools and the remainder were recruited from Catholic and independent schools (eTable 2 in Supplement 2). Over the course of the study (2013-2015), all schools remained enrolled (Figure).

**Figure.**
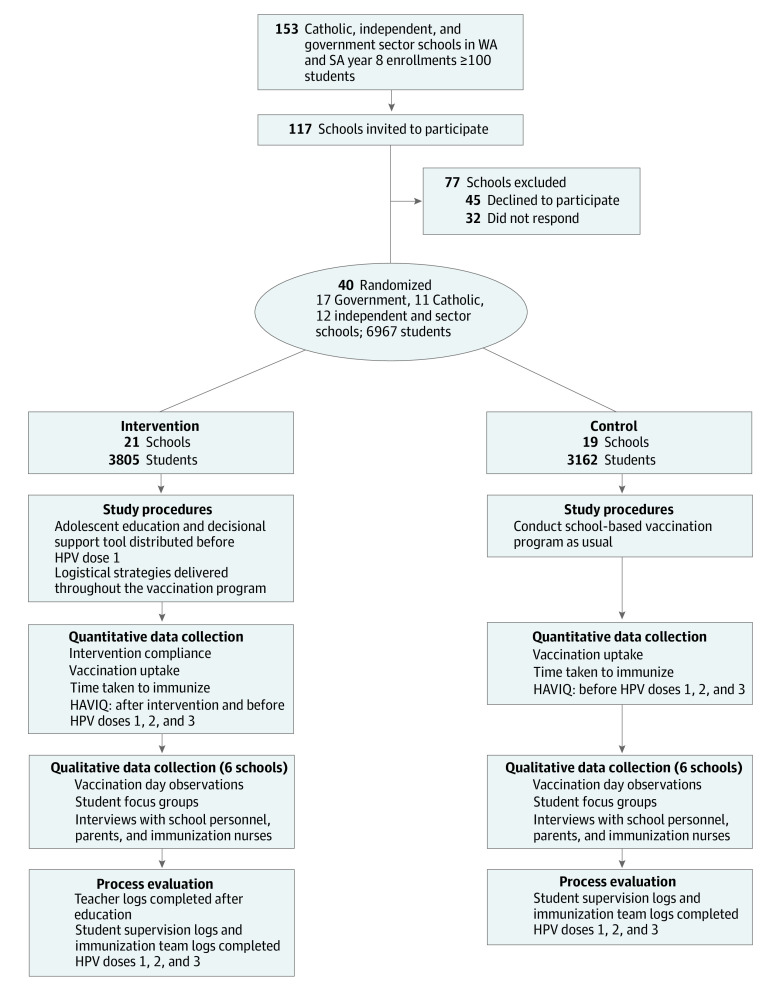
HPV.edu Study Flow Diagram HAVIQ indicates HPV Adolescent Vaccination Intervention Questionnaire; HPV, human papillomavirus; SA, South Australia; WA, Western Australia.

### HAVIQ Response Rates

The overall response rate for the HAVIQ questionnaire was 55%. In WA, where parental consent was required, the response rate was 35% (1676 of 4751 students), and in SA, where parental consent was not required, it was 97% (2166 of 2216 students). HAVIQ scores were adjusted for year, state, school type, and clustering of students within schools (eTable 3 in Supplement 2).

### Adolescent HPV Decision-making

Table 1 shows adolescent HPV vaccination decision-making scores. The mean (SD) score before dose 1 for decision-making on the HAVIQ subscale 2 was 3.50 (0.42) of 5 points in the intervention group and 3.40 (0.40) in the control group, a small but statistically significant difference of 0.11 point (95% CI, 0.06-0.16 point; *P* < .001).

**Table 1.** Adolescent HPV Vaccination Decision-making (HPV Adolescent Vaccination Intervention Questionnaire Subscale 2) Before Dose 1

Group	Students with valid scores, No.	Score, mean (SD)^a^	Difference (95% CI)^b^	*P* value^b^
Intervention	1682	3.50 (0.42)	0.11 (0.06-0.16)	<.001
Control	1401	3.40 (0.40)		

Abbreviation: HPV, human papillomavirus.

^a^ Mean score is calculated as the mean of 8 decision-making questions. Responses to questions on a Likert scale range from 1 (strongly disagree) to 5 (strongly agree), with higher scores indicating more individual decision-making.

^b^ Adjusted for year, state, sector, coeducational status, and clustering of students within schools.

### Adolescent Vaccination-Related Anxiety Scores

There was a small reduction in vaccination-related anxiety in the intervention group compared with the control group (Table 2). Both groups became less anxious with each dose of the vaccine (pre–dose 1 difference, −0.11 point [95% CI, −0.19 to −0.02 point]; pre–dose 2 difference, −0.18 point [95% CI, −0.26 to −0.10 point]). Before dose 3, the mean (SD) scores for HAVIQ subscale 3 were 2.3 (0.82) of 5 points in the intervention group and 2.5 (0.80) in the control group, a difference of −0.18 point (95% CI, −0.24 to −0.11 point; *P* < .001).

**Table 2.** Adolescent HPV Vaccination-Related Fear and Anxiety (HPV Adolescent Vaccination Intervention Questionnaire Subscale 3)

Time and group	Students with valid fear and anxiety scores, No.	Score, mean (SD)^a^	Difference (95% CI)^b^	*P* value^b^
Before dose 1				
Intervention	1713	2.6 (0.82)	−0.11 (−0.19 to −0.02)	.008
Control	1412	2.7 (0.82)		
Before dose 2				
Intervention	1795	2.4 (0.83)	−0.18 (−0.26 to −0.10)	<.001
Control	1365	2.6 (0.86)		
Before dose 3				
Intervention	1729	2.3 (0.82)	−0.18 (−0.24 to −0.11)	<.001
Control	1493	2.5 (0.80)		

Abbreviation: HPV, human papillomavirus.

^a^ Mean score is calculated as the mean for 6 fear and anxiety questions. Responses to questions on a Likert scale range from 1 (strongly disagree) to 5 (strongly agree), with lower scores indicating less fear and anxiety.

^b^ Adjusted for year, state, sector, coeducational status, and clustering of students within schools.

### Adolescent Vaccination Self-efficacy and Skills

There was a small increase in vaccination-related self-efficacy in the intervention group compared with the control group (Table 3). Both groups became more confident with each dose of the vaccine (pre–dose 1 difference, 4.0 points; [95% CI, 1.0-7.0 points]; pre–dose 2 difference, 4.0 points [95% CI, 2.0-6.0 points]). Before dose 3, the mean (SD) scores for HAVIQ subscale 4 were 84.0 (18.2) of 100 for the intervention group and 80.0 (18.8) for the control group, an adjusted mean difference of 3.0 points (95% CI, 1.0-5.0 points; *P* = .002).

**Table 3.** Student HPV Vaccination Self-efficacy and Skills (HPV Adolescent Vaccination Intervention Questionnaire Subscale 4)

Time and group	Students with valid scores, No.	Score, mean (SD)^a^	Difference (95% CI)^b^	*P* value^b^
Before dose 1				
Intervention	1727	74.0 (20.4)	4.0 (1.0-7.0)	.006
Control	1443	71.0 (21.2)		
Before dose 2				
Intervention	1802	81.0 (18.7)	4.0 (2.0-6.0)	<.001
Control	1362	76.0 (20.5)		
Before dose 3				
Intervention	1757	84.0 (18.2)	3.0 (1.0-5.0)	.002
Control	1538	80.0 (18.8)		

Abbreviation: HPV, human papillomavirus.

^a^ Mean score is calculated as the mean for 5 skills questions. Responses to questions range from 0 to 100 with higher scores indicating more skills and confidence.

^b^ Adjusted for year, state, sector, coeducational status, and clustering of students within schools.

### Qualitative Findings of Student Psychosocial Outcomes

#### HPV Vaccine Decision-making

Participants from the intervention group reported greater involvement in HPV vaccine decision-making and discussion with parents, informed by increased knowledge, understanding, and self-advocacy (“I just told my parents and then they didn’t want me to do it at first, but I told them like what it does and that it could prevent [cancer]”). In contrast, control group participants reported no or reduced engagement in HPV decision-making because they had no or limited information before vaccination commenced (“If we don’t know anything then it is really just another injection”). Lack of information resulted in varied adolescent responses, including compliance (“So, basically I really didn’t have a choice”), support (“It is in the best interests of your child”), or anger (Researcher: “And how did you feel about it?” Adolescent: “Mad”). Some adolescents in the control group reported that if they were the primary vaccination decision-makers, they would refuse the vaccine because of fear of needles (“I think if they [adolescents] had a choice they would say no”) and no understanding of the benefits of the vaccine (“our class we didn’t know what they [vaccines] were necessarily for”), whereas some adolescents in the intervention group wanted “some sort of system” for medical professionals to enable adolescents to consent for their own vaccination (Table 4).

**Table 4.** Focus Group Findings for 111 Adolescents

Theme	Control^a^	Intervention^a^
Adolescent involvement in HPV vaccine decision-making	“If we know what it [the HPV vaccine] is, what is it going to do for us…But if we don’t know anything then it is really just another injection.”	“I just told my parents and then they didn’t want me to do it at first, but I told them like what it does and that it could prevent [cancer].”
	“I think if they [adolescents] had a choice they would say no…We didn’t know what they were necessarily for.”	“I kind of thought it would be dumb not to do it. So, it was kind of not a kind of choice. Yeah, not a really smart thing not to do it.”
	“Basically, you just showed up at school one day and they said you are getting it, the vaccination. So, basically, I really didn’t have a choice.”	“If the parent says ‘no,’ then there should be some sort of system where the medical people can analyze what the situation is and like how much the child might be…how important it is for them.”
		“I think I probably would have got it anyway, but I wanted to get it. Like there was no point in risking it.”
Adolescent support of parents’ HPV vaccine decision-making	“Every parent wants their child to grow up healthy. It is in the best interests of your child so it is a good idea that you do it even if the child doesn’t necessarily want to do it, it will be better for them in the long run.”	“I think my parents know best for me. They have obviously thought about it lot more than we have.”
Mother made HPV vaccine decision more frequently than father	“My mother actually decided for me to get the HPV vaccination.” Researcher: “Did she discuss that with you?” “Not really.” Researcher: “How did you feel about it?” “Mad.”	“After watching the video [at school] I told Mum that we watched it and when we got the note she came and just went through, and she signed the note…I told a bit more of what I had learnt.”
	“My Mum kind of…she said you should get it.”	“My dad’s relationship with me is different to…I was like, yeah, I am going to have these needles, and he was like, oh that’s okay and I’m like this is going to help me if this [cervical cancer] happens to me.”
Adolescent anxiety		
Needles	“I think I will always be scared when I get them…I get scared when I watch other people get needles…I just look away.”	“If we weren’t good with needles, they told us how to stay calm during it. So that was fine.”
	“The thing that scared me was like when I walked in and my injection was sitting there ready.”	“A lot of people were really nervous about getting it. The first one [HPV dose 1].”
	“I was really scared because like needles really scare me but…It hurts, yeah. But once I got it, it was fine. I felt the second one [vaccine] more…Yeah. I mean it really hurt…and kind of like an aching pain.”	“I think it is good like if one of your friends is there to like they can talk to you and like calm you down. Like I was really stressed out because I don’t like needles.”
	“We had our tetanus and the tetanus hurt so I guess when you had the HPV it didn’t hurt as much which was good. The last one was the worst out of the three vaccinations for HPV. [The vaccine had]…a larger dose in.”	“I find that when I get vaccines because I get anxious about it, I have to lie down because I get a bit dizzy.”
		“I don’t really mind if it is just one, I don’t mind, but two!”
Distraction	“Give us something to do in the line because I think like most of the time you are really bored.”	“They had like iPads for us to play on, to get distracted. [The iPads] talked about the virus as well.”
	“The person injecting you made it really easy because they had like a conversation with you. They take your mind off it and they put it [the vaccine] in.”	“They [nurses] are used to dealing with kids everywhere, so they [say] like it might hurt a little bit. Yeah, but it will be over in like a minute.”
Rumors, exaggeration	“Like [other students] over exaggerate and try and tense them up and try to show that they are tough.”	“Sometimes the people that are like stressing you out because they are saying like, they will make up stuff and or like they are really scared about it, so we try and help them, and they are like not calming down and then we have to put more effort into calming them down.”
	“There was like a rumor going around at the start of it that…just the vaccine because there was like it was new and stuff and they were just testing it out on the public school kids because they are more disposable than the private school kids.”	“And some people were like…they overexaggerate it…We kind of like dramatize everything, people…get really scared and like me, I really could not care less about getting an injection. Like an ordinary day, I wouldn’t care.”
	“Before we get it [the vaccine] like everybody is like really…does it hurt and then somebody says…they started bleeding a lot!…No one really tells you anything. You don’t know.”	
	“I just told everyone to just try and freak everyone out…Just…say oh it is gonna hurt…and they get freaked out…She [another student] cried.”	
	“Everyone was like freaking out…Because the other classes, because we were the last class to have ours, and because everyone else is coming back and they are saying, oh they are so sore.”	
Fainting	“My friend fainted like not this time but the last time. So, I think he was pretty anxious.”	“I half fainted…Yeah, I was about to faint and then the nurses had to put me down on a mattress…It was a bit awkward really. I wasn’t expecting to faint…And my eyes like rolled back a little bit…I have heard that it happens to most kids and stuff. But I was just yeah worried about me hitting my head on something.”
	[Adolescent fainted between administration of 2 vaccines]. “I fainted the first time. Um, just after they gave me the second last [vaccine], I was feeling a bit dizzy, so they made me lie on the ground and I fainted.”	“I fainted, and I threw up too…A couple of kids cried and stuff because they were worried. But after it was done, they said it was pretty good.”
Embarrassment, vaccination environment and logistics	“I don’t think some people in the room liked it [being visible to peers during vaccination]. Maybe the person who was getting the vaccination—they might have felt a bit uncomfortable, yeah. Embarrassed…I saw them crying.”	“There are people who actually like genuinely scared. Yeah. Generally, they are the ones who don’t really cry, they are just scared…Like once they get really hysterical you know it has a follow-on effect on people.”
	Researcher: “When you were being vaccinated could other students see you being vaccinated?” “Yeah.” Researcher: “And how did you feel about that?” “The first time I was scared that I was going to cry.”	“You stand in a line…and then they make you go in like two at a time. But then…[you] have to stand back because you can’t be looking in and laughing at them.”
	“There were lots of kids coming out crying, it was kind of disturbing.”	Researcher: “Could people see you getting vaccinated?” “No. Not really.”
Self-efficacy (confidence)	“I didn’t know what I was getting.”	“I learnt everything [at school]…I learnt that it existed, which is very important, I think.”
	“I think that for me, because I personally get like scared of having injections. If I knew it was actually something that was like helpful, then it would make me think differently about it and feel more like better about having an injection.”	“It is really important for people to know about it.”
	“I don’t think there would be much of a risk with taking vaccinations because the people who made them know what they are doing.”	“When you actually get your needle done, everyone is really nice to you and it is really calming and you have got support around you, so it is okay…And so, it is a good feeling on the day.”
	[HPV dose 2] “Probably easier because then you know what it was like. You knew what to expect.”	“People were getting scared and I am just…it is really effective, and it is done.”
	“I remember I was sitting and waiting and then I heard someone scream when they had theirs and it really freaked me out.”	“Just keeping it going in school because, as I said, it is an experience…I think it is part of going to school.”
		[HPV dose 2-3] “Because you get used to it already, so then you kind of know how much it is going to actually hurt. So then, yeah, we just prepare ourselves.”
		“I want to be protected for later in life.”

Abbreviation: HPV, human papillomavirus.

^a^ All quotations are from adolescents unless otherwise noted.

#### Adolescent Vaccination-Related Fear and Anxiety

Adolescents in both groups reported needle-related anxiety, especially at HPV dose 1, with heightened anxiety while awaiting vaccination and having more than 1 vaccine administered simultaneously (“I don’t really mind if it is just one…but two!”). The intervention group reported that study iPads helped to distract them while awaiting vaccination (“They had like iPads for us to play on, to get distracted”), whereas the control group wanted to be distracted. Both groups reported immunization nurses were skilled at distracting them during vaccination. The control group compared the pain associated with HPV vaccination to that of diphtheria-tetanus-acellular pertussis vaccination.

The intervention group reported self-moderating needle-related fear and anxiety on vaccination day as a result of their HPV vaccination knowledge. Both study groups reported rumors about the vaccination experience; however, rumors were less prominent in intervention group data than in control (“They were just testing it [HPV vaccine] out on the public-school kids” [control]). Both groups reported concerns about crying in front of peers. A few students across both study groups reported fainting or vomiting on vaccination day. Some adolescents in the control group reported seeing and hearing peers getting vaccinated, which increased anxiety for those awaiting vaccination (“I saw them crying” [control]). Adolescents in the intervention group reported being discouraged by teachers from watching or embarrassing their peers during vaccination. Many in the intervention group reported that they were unable to see their peers vaccinated because of the room setup. The intervention group reported increased peer support on vaccination day (“we have to put more effort into calming them down”) (Table 4).

#### Adolescent Vaccination-Related Self-efficacy

Control school adolescents reported less confidence on vaccination day than the intervention group (“I personally get like scared of having injections” [control]). The intervention group described how their confidence in vaccination was supported by (1) knowledge about HPV vaccination, (2) skills and activities used to stay calm and distraction on vaccination day, (3) normalization of vaccination and peer support in the school setting, (4) experience of vaccination after HPV dose 1, and (5) trust in HPV vaccination experts. In contrast, the control group reported low confidence because many had limited or no knowledge about HPV vaccination. However, the control group described that their confidence in vaccination was supported by normalization of vaccination and peer support in the school setting and trust in HPV vaccination experts (Table 4).

## Discussion

In this analysis of secondary outcomes of a cluster randomized trial, both quantitative and qualitative data indicated that our intervention improved adolescent psychosocial outcomes, although the overall differences were small. The intervention also increased adolescents’ knowledge about HPV, vaccination, and the vaccination process.^27^ This outcome, in turn, promoted vaccination literacy at a time when adolescent cognitive, physical, and emotional processes and health-related behaviors are developing.^33^ Adolescent participation in vaccine decision-making and discussion with parents increased after our educational intervention at school about HPV vaccination. Adolescents in the control group who were uninformed about HPV vaccination found participation in decision-making difficult because they had low or no knowledge about HPV vaccines.

An ecological framework is useful to understand the psychological, social, and organizational levels of influence on adolescents within a school vaccination system.^32^ Although much research has focused on health care practitioner recommendation^34,35,36,37,38^ and parent HPV vaccine decision-making for their adolescents,^39,40,41^ less attention has been dedicated to shared vaccination decision-making and informative discussion with adolescents.^4,42,43,44^ However, these factors can help promote healthy behaviors and adolescent autonomy and contribute to efficient health service provision on vaccination day, leading to more confident, vaccination-literate adolescents.^2,45^ Adolescents are frequently excluded from HPV vaccine decision-making because of their age, limited adolescent and parent knowledge about HPV vaccination, logistical barriers associated with the vaccination setting, and communication challenges between parents, adolescents, and health care practitioners.^4,10,42,44^

The school classroom is conducive to active adolescent engagement about HPV vaccination.^2,26,27,46,47^ Before vaccination, in-school education such as our intervention about HPV vaccination can promote adolescent involvement in decision-making and increase self-efficacy based on accurate knowledge and understanding. In-school education about HPV vaccination may have utility beyond the school setting, such as being well-informed before attending vaccination in a traditional clinical setting. Health and education sectors can work together to better meet the challenge of an adolescent vaccination literacy deficit^33^ and to improve the adolescent vaccination experience.

Our intervention recognized adolescents as active partners in decision-making about their health because, throughout the research process, we used integrated knowledge translation that included codesigning evidence-based resources with youth and experts.^48^ Adolescent health literacy, including information about rights to make health decisions, is important to strengthen young people’s knowledge, motivation, and competency to make well-informed health decisions in their own best interests, with involvement of their parents as appropriate.^44,49,50^ Qualitative data showed that our intervention increased discussion with parents about receiving the vaccine, with some adolescents in the intervention group advocating to their parents and peers about the vaccine. Adolescents involved in discussions about HPV vaccination with a parent viewed the vaccination decision as mutual, regardless of the parent’s opinion.^45^ Adolescents in the intervention group requested that health care systems and professionals incorporate methods to assess and implement adolescent self-consent to vaccination.^49^

Needle-related fear, which can result in behavioral distress and vasovagal syncope, is experienced on a continuum from anxiety related to needles to needle phobia.^51,52^ Here, we found that adolescents in the intervention group experienced higher confidence on vaccination day after HPV vaccination education. Improved vaccination literacy enabled adolescents to rationalize and tolerate the short-term discomfort of HPV vaccination because they understood the long-term benefits of HPV-related cancer prevention. Adolescent needle fear can have its origins in childhood and is associated with lower HPV vaccination initiation in adolescence.^53^

Adolescent needle fear and anxiety can also be influenced by the vaccination setting. The school presents circumstances different from a clinic setting, including the absence of parents, presence of peers and school personnel, and conversion of the school setting into a temporary clinic.^17^ The school setting expects adolescents to be much more autonomous and self-regulated and to use effective management strategies suited to their developmental stage and to the vaccination setting.^17^ Factors associated with a positive vaccination day experience for adolescents, such as those in our logistical intervention, include privacy (eg, screens and separate entrance and exit to the school clinic to prevent students mixing before and after vaccination), vaccinating anxious adolescents first, ensuring that adolescents waiting for vaccination are in small groups and have access to distraction,^54^ and vaccinating adolescents early in the day when possible. In addition, educating adolescents about HPV, HPV vaccination, and useful distraction techniques at school before vaccination can reduce needle-related fear and anxiety, increase self-efficacy on vaccination day, and assist in addressing rumors and vaccine hesitancy.^10^

### Limitations

Generalizability of these findings may be limited for several reasons. First, although this was a stratified random sample of schools, just over one-third of schools agreed to participate. Second, it is possible that there was a selection bias toward schools more interested in vaccination and/or research, which could decrease the generalizability of the intervention’s impact to all schools.^27^ Third, schools were not blinded to the intervention group. Fourth, the response rate to the HAVIQ was only 55%. In addition, as previously reported,^27^ there was no effect of the complex intervention on the primary outcome of vaccination uptake. Fifth, the differences we observed of the educational intervention on psychosocial outcomes over the vaccination course, although significant, were small, and further research is needed to determine whether they represent an important difference.

## Conclusions

School-based vaccination provides unique opportunities that help support the adolescent vaccination experience. In this cluster randomized trial, the intervention in the school setting promoted adolescent vaccination health literacy and decisional involvement and vaccine-related confidence and reduced needle-related fear and anxiety that were maintained throughout the vaccination course. Improved health literacy, combined with logistical strategies, resulted in improved vaccination experiences for adolescents in the school setting. Guidelines for school-based vaccination should incorporate advice regarding how this outcome can be achieved.

## Notes

Supplement 1.
Trial Protocol

Supplement 2. **eTable 1.** Timing for Measurement of Outcomes **eTable 2.** Summary of Participating Schools and Students **eTable 3.** Questionnaire Response Rates

Supplement 3.
HPV.edu Study Group Members

Supplement 4.
Data Sharing Statement
