# Supplementary material for: Effect of a School-Based Educational Intervention About the Human Papillomavirus Vaccine on Psychosocial Outcomes Among Adolescents: Analysis of Secondary Outcomes of a Cluster Randomized Trial
Source: JAMA Netw Open. 2021 Nov 2;4(11):e2129057. doi: 10.1001/jamanetworkopen.2021.29057 (PMC8564580; doi:10.1001/jamanetworkopen.2021.29057)
Supplement: Supplement 2. — eTable 1. Timing for Measurement of Outcomes eTable 2. Summary of Participating Schools and Students eTable 3. Questionnaire Response Rates [file jamanetwopen-e2129057-s002.pdf]

Supplemental Online Content

Davies C, Marshall HS, Zimet G, et al; HPV.edu Study Group. Effect of a school-based educational intervention about the human papillomavirus vaccine on psychosocial outcomes among adolescents: analysis of secondary outcomes of a cluster randomized trial. *JAMA Netw Open*. 2021;4(11):e2129057. doi:10.1001/jamanetworkopen.2021.29057

eTable 1. Timing for Measurement of Outcomes
eTable 2. Summary of Participating Schools and Students
eTable 3. Questionnaire Response Rates

This supplemental material has been provided by the authors to give readers additional information about their work.

© 2021 Davies C et al. *JAMA Network Open.*

eTable 1: Timing for Measurement of Outcomes

**Outcome**	**Pre-HPV dose 1**	**HPV dose 1**	**Pre-HPV dose 2**	**HPV dose 2**	**Pre-HPV dose 3**	**HPV dose 3**
**Knowledge**	B				B	
**Attitudes**	B				B	
**Decision-making**	B					
**Fear/Anxiety**	B		B		B	
**Self-efficacy**	B		B		B	

B = Measured in both intervention and control schools.
Thick vertical red line = Intervention begins.

© 2021 Davies C et al. *JAMA Network Open.*

eTable 2: Summary of Participating Schools and Students

		Intervention			Control	
		Schools (n (%))	Students enrolled in participating years (n(%))		Schools (n(%))	Students enrolled in participating years (n(%))
Total		21	3806		19	3162
Year	2013	4 (19)	554 (15)		3 (16)	450 (14)
	2014	17 (81)	3251 (85)		16 (84)	2712 (86)
State	South Australia	8 (38)	1162 (31)		8 (42)	1054 (33)
	Western Australia	13 (62)	2643 (69)		11 (58)	2108 (67)
Sector	Government	9 (43)	2042 (54)		8 (42)	1488 (47)
	Independent	7 (33)	979 (26)		5 (26)	648 (20)
	Catholic	5 (24)	784 (21)		6 (32)	1026 (32)
Co-educational	Yes	16 (76)	3082 (81)		15 (79)	2530 (80)
	Female only	2 (10)	245 (6)		2 (11)	248 (8)
	Male only	3 (14)	478 (13)		2 (11)	384 (12)
Total enrolled in school	< 800	2 (10)	196 (5)		4 (21)	401 (13)
	800 to 999	10 (48)	1557 (41)		3 (16)	442 (14)
	1000 to 1299	2 (10)	343 (9)		9 (47)	1763 (56)
	1300 and over	7 (33)	1709 (45)		3 (16)	556 (18)
ICSEA group	< 1000	4 (19)	975 (26)		2 (11)	270 (9)
	1000 to 1049	6 (29)	934 (25)		5 (26)	823 (26)
	1050 to 1099	4 (19)	666 (18)		7 (37)	1380 (44)
	1100 and over	7 (33)	1230 (32)		5 (26)	689 (22)
Previous vaccination rate	≤ 70%	5 (24)	999 (26)		3 (16)	417 (13)
	71% to 80%	8 (38)	1736 (46)		5 (26)	958 (30)
	81% to 85%	5 (24)	719 (19)		6 (32)	1051 (33)
	> 85%	2 (10)	247 (6)		4 (21)	563 (18)
	No previous vaccinations at school	1 (5)	104 (3)		1 (5)	173 (5)

© 2021 Davies C et al. *JAMA Network Open.*

	1100 and over	7 (33)	1230 (32)	1294 (75.7)		5 (26)	689 (22)	446 (80.2)
Previous vaccination rate	≤ 70%	5 (24)	999 (26)	701 (70.2)		3 (16)	417 (13)	273 (65.5)
	71% to 80%	8 (38)	1736 (46)	1287 (74.1)		5 (26)	958 (30)	752 (78.5)
	81% to 85%	5 (24)	719 (19)	583 (81.1)		6 (32)	1051 (33)	860 (81.8)
	> 85%	2 (10)	247 (6)	208 (84.2)		4 (21)	563 (18)	465 (82.6)
	No previous vaccinations at school	1 (5)	104 (3)	91 (87.5)		1 (5)	173 (5)	133 (76.9)

© 2021 Davies C et al. *JAMA Network Open.*

eTable 3: Questionnaire Response Rates

Group	Schools (n)	Students enrolled in WA	Students in WA completing q'naire (%)	Students enrolled in SA	Students in SA completing q'naire (%)	Total Students enrolled	Total students completing q'naire (%)
**Intervention**	21	2643	906 (34%)	1162	1136 (98%)	3805	2042 (53%)
**Control**	19	2108	770 (37%)	1054	1030 (98%)	3162	1800 (57%)
**Total**	40	4751	1676 (35%)	2216	2166 (98%)	6967	3842 (55%)

© 2021 Davies C et al. *JAMA Network Open.*
