# Supplementary material for: Effect of a School-Based Educational Intervention About the Human Papillomavirus Vaccine on Psychosocial Outcomes Among Adolescents: Analysis of Secondary Outcomes of a Cluster Randomized Trial
Source: JAMA Netw Open. 2021 Nov 2;4(11):e2129057. doi: 10.1001/jamanetworkopen.2021.29057 (PMC8564580; doi:10.1001/jamanetworkopen.2021.29057)
Supplement: Supplement 3. — HPV.edu Study Group Members [file jamanetwopen-e2129057-s003.pdf]

Supplemental Online Content: Nonauthor Collaborators

*Indicates required information. Only first name, last name, and suffix will appear in PubMed.

*Group Name(s): HPV.edu Study Group							
***First Name and Middle Initial(s)**	***Last Name**	***Suffix (eg, Jr, III)**	Academic Degrees	Institution	Location (city, state/province, country)	Role or Contribution, eg, chair, principal investigator	Group (if more than 1 Group listed in the byline) and/or Subgroup (eg, Steering Committee)
Annette	Braunack-Mayer		PhD	Australian Centre for Health Engagement, Evidence and Values, School of Health and Society, Faculty of Arts, Social Sciences and Humanities, University of Wollongong	NSW, Australia	Associate Investigator	HPV.edu study group
Joanne	Collins		PhD	Women's and Children's Health Network and School of Medicine and Robinson Research Institute, University of Adelaide, SA, Australia	SA, Australia	Research study staff	HPV.edu study group
Spring	Cooper		PhD	School of Public Health, City University of New York (CUNY)	New York, NY, USA	Chief Investigator	HPV.edu study group
Heidi	Hutton		M.Sc.	Telethon Kids Institute, University of Western Australia	WA, Australia	Research study staff	HPV.edu study group
Jane	Jones		Bachelor of Environmental Science, Bachelor of Nursing	Telethon Kids Institute, University of Western Australia	WA, Australia	Research study staff	HPV.edu study group
Julie	Leask		PhD	Susan Wakil School of Nursing and Midwifery, Faculty of Medicine and Health, University of Sydney	NSW, Australia	Associate Investigator	HPV.edu study group

Supplemental Online Content: Nonauthor Collaborators

*Indicates required information. Only first name, last name, and suffix will appear in PubMed.

*First Name and Middle Initial(s)	*Last Name	*Suffix (eg, Jr, III)	Academic Degrees	Institution	Location (city, state/province, country)	Role or Contribution, eg, chair, principal investigator	Group (if more than 1 Group listed in the byline) and/or Subgroup (eg, Steering Committee)
Adriana	Parrella		PhD	Women's and Children's Health Network and School of Medicine and Robinson Research Institute, University of Adelaide; and South Australian Health and Medical Research Institute (SAHMRI), Adelaide, Australia	SA, Australia	Research study staff	HPV.edu study group
David G.	Regan		PhD	The Kirby Institute for Infection and Immunity in Society, Faculty of Medicine, UNSW	NSW, Australia	Chief Investigator	HPV.edu study group
Peter	Richmond		MBBS MRCP (London) FRACP	Perth Children's Hospital, Child and Adolescent Health Service, Western Australia, Wesfarmers Centre of Vaccines and Infectious Diseases, Telethon Kids Institute, WA, Australia, and School of Medicine, University of Western Australia	WA, Australia	Associate Investigator	HPV.edu study group
Tanya	Stoney		MBBS	Telethon Kids Institute, University of Western Australia	WA, Australia	Chief Investigator	HPV.edu study group
