# Supplementary material for: Effect of a School-Based Educational Intervention About the Human Papillomavirus Vaccine on Psychosocial Outcomes Among Adolescents: Analysis of Secondary Outcomes of a Cluster Randomized Trial
Source: JAMA Netw Open. 2021 Nov 2;4(11):e2129057. doi: 10.1001/jamanetworkopen.2021.29057 (PMC8564580; doi:10.1001/jamanetworkopen.2021.29057)
Supplement: Supplement 4. — Data Sharing Statement [file jamanetwopen-e2129057-s004.pdf]

Data Sharing Statement

Davies. Effect of a School-Based Educational Intervention About the Human Papillomavirus Vaccine on Psychosocial Outcomes Among Adolescents. *JAMA Netw Open*. Published November 02, 2021. doi:10.1001/jamanetworkopen.2021.29057

Data
Data available: No

Additional Information
Explanation for why data not available: HPV.edu study data includes immunisation data that belongs to state jurisdictions and cannot be shared.
